# Minimally Invasive Percutaneous Pedicle Screw Fixation in Thoracolumbar Spine Fractures: A Clinical Evaluation Study and Computed Tomography Scan Analysis of Screw Tracts

**DOI:** 10.7759/cureus.92065

**Published:** 2025-09-11

**Authors:** Nidhil Noushad, Hariprasad Seenappa

**Affiliations:** 1 Orthopaedics, Sri Devaraj Urs Medical College, Sri Devaraj Urs Academy of Higher Education and Research, Kolar, IND

**Keywords:** computed tomography, functional outcomes, minimally invasive surgery, pedicle screws, percutaneous fixation, spine fractures, thoracolumbar spine

## Abstract

Background and objective

Thoracolumbar spine fractures represent significant traumatic injuries with substantial morbidity potential, necessitating surgical interventions that balance stability with minimal tissue disruption. Conventional open approaches, while providing excellent visualization, are associated with considerable muscle damage, blood loss, and prolonged recovery periods. Minimally invasive percutaneous pedicle screw fixation (MIPPSF) has emerged as a promising alternative that theoretically reduces surgical trauma while maintaining effective stabilization. This prospective interventional study aimed to evaluate the functional outcomes of MIPPSF among patients with thoracolumbar spine fractures and analyze screw tract placement accuracy using computed tomography scanning in carefully selected patients without neurological deficits.

Methodology

This prospective interventional study was conducted from May 2023 to October 2024 at R.L. Jalappa Hospital and Research Centre, Tamaka, India. Thirty patients presenting with thoracolumbar spine fractures requiring dorsal instrumentation were enrolled. Inclusion criteria encompassed patients >18 years with thoracic or lumbar spine fractures secondary to trauma, spinal neoplasia, or degenerative disorders without neurological deficits. Exclusion criteria comprised retropulsion segments occupying >50% of the spinal canal, previous spine surgery at the same level, significant posttraumatic segmental kyphosis ≥10°, and fracture dislocations. Functional outcomes were systematically assessed using validated instruments: Visual Analog Scale (VAS), Straight Leg Raise Test (SLRT), Modified Oswestry Disability Index (MODI), Core Outcome Measures Index (COMI), and Roland-Morris Questionnaire (RMQ). Assessments were performed preoperatively and at postoperative intervals of one, three, and six months. Radiological evaluation included postoperative CT scan analysis to identify screw tracts and potential pedicle breaches.

Results

Anatomical distribution revealed predominance at the L1-L2 intervertebral region (n= 18, 60.0%). All functional parameters demonstrated statistically significant improvement across successive timepoints (p<0.001). VAS pain scores decreased progressively from baseline (mean=7.83, SD=0.69) to six months postoperatively (mean=1.77, SD=0.43), representing a 77.4% reduction. SLRT improved from baseline (mean=49.67°, SD=8.08) to six months (mean=75.67°, SD=7.73), indicating 52.3% functional enhancement. MODI scores demonstrated a substantial reduction from baseline (mean=75.47, SD=7.06) to six months (mean=5.60, SD=1.99), reflecting a 92.6% decrease in disability. COMI scores improved from baseline (mean=6.35, SD=1.17) to six months (mean=1.98, SD=0.75), showing 68.8% improvement. RMQ scores decreased from baseline (mean=20.20, SD=1.95) to six months (mean=3.53, SD=1.71), representing an 82.5% reduction. Radiological assessment revealed exceptional accuracy, with only one patient (3.3%) exhibiting lateral pedicle breach, resulting in a 96.7% accuracy rate.

Conclusion

MIPPSF demonstrates excellent clinical and radiological outcomes in carefully selected patients with thoracolumbar fractures. Progressive functional improvement across all parameters, coupled with high screw placement accuracy, supports MIPPSF as an effective therapeutic option, achieving significant pain reduction and functional recovery while maintaining technical precision.

## Introduction

The thoracolumbar junction represents a critical biomechanical transition between the relatively rigid thoracic spine and the more mobile lumbar region, making it particularly susceptible to traumatic injuries. Approximately 90% of all spinal fractures occur in the thoracolumbar region, with the majority affecting the T11-L2 vertebrae due to their unique biomechanical properties and transitional anatomy [1,2]. Management of thoracolumbar fractures has evolved substantially over recent decades, with treatment paradigms shifting toward more minimally invasive approaches that aim to reduce iatrogenic tissue trauma while maintaining or improving clinical outcomes.

Conventional open surgical techniques for thoracolumbar fracture fixation, while effective, are associated with significant soft tissue disruption, substantial blood loss, prolonged hospitalization, and extended rehabilitation periods [3,4]. These limitations have prompted the development of minimally invasive surgical (MIS) techniques, particularly percutaneous pedicle screw fixation, which has emerged as a viable alternative for treating thoracolumbar fractures without neurological deficits [5,6]. The percutaneous approach facilitates the insertion of pedicle screws through small paramedian incisions under fluoroscopic guidance, preserving the integrity of paraspinal musculature and reducing surgical morbidity [7].

The theoretical advantages of minimally invasive percutaneous pedicle screw fixation include reduced blood loss, diminished postoperative pain, decreased infection rates, shorter hospitalization, and accelerated functional recovery [8,9]. Gong et al. demonstrated that percutaneous techniques significantly attenuate inflammatory responses compared to conventional open procedures, potentially contributing to reduced postoperative pain and enhanced recovery trajectories [10]. Furthermore, meta-analyses have consistently reported comparable or superior outcomes regarding radiological parameters, functional improvement, and complication rates with minimally invasive approaches compared to traditional open surgery [11,12].

Despite these promising results, the accurate placement of pedicle screws via percutaneous techniques remains technically demanding and heavily dependent on intraoperative imaging [13]. Traditional fluoroscopy-guided techniques, while widely accessible, pose challenges in achieving optimal three-dimensional visualization of complex vertebral anatomy. Consequently, technological innovations, including CT navigation systems, electromagnetic navigation, and robotic assistance, have been integrated to enhance the precision of screw placement and reduce radiation exposure [14,15]. Kim et al. reported that intraoperative CT guidance significantly improved the accuracy of pedicle screw placement compared to conventional fluoroscopy, with malposition rates of 5.3% and 15%, respectively [16].

The radiological assessment of screw tract morphology and pedicle breach presents a critical component in evaluating the technical success of percutaneous instrumentation. Computed tomography provides superior visualization of the osseous anatomy and offers precise evaluation of screw positioning relative to the pedicle and adjacent neurovascular structures [17]. Winder and Gilhooly utilized three-dimensional CT analysis to retrospectively assess screw placement accuracy, demonstrating that percutaneous techniques achieved acceptable positioning in 94.8% of inserted screws, with clinically significant breaches occurring in only 2.4% of cases [18].

While the literature increasingly supports the efficacy of minimally invasive percutaneous pedicle screw fixation (MIPPSF) for thoracolumbar fractures, significant knowledge gaps persist regarding the correlation between radiological parameters of screw positioning and functional outcomes. Additionally, the long-term sustainability of correction parameters and hardware-related complications remains incompletely characterized [19]. To address these limitations, this prospective interventional study aims to comprehensively evaluate the functional outcomes of MIPPSF and conduct a detailed radiological analysis of screw tracts using computed tomography in patients with thoracolumbar spine fractures.

## Materials and methods

Study design and study setting

This investigation was designed as a prospective interventional study to evaluate the functional outcomes and radiological accuracy of MIPPSF in thoracolumbar spine fractures. All clinical procedures and patient evaluations were conducted at the Department of Orthopaedics, R.L. Jalappa Hospital and Research Centre, Tamaka, India, which is affiliated with Sri Devaraj Urs Medical College and Sri Devaraj Urs Academy of Higher Education and Research, Kolar, India.

Study period

The study was conducted over a 17-month period from May 2023 to October 2024.

Ethics committee approval

Prior to initiating patient recruitment, comprehensive ethical clearance was obtained from the Institutional Ethics Committee of Sri Devaraj Urs Medical College (approval number: DMC/KLR/IEC/80/2023-24). The study protocol adhered to the ethical principles outlined in the Declaration of Helsinki and was conducted in accordance with Good Clinical Practice guidelines. Written informed consent was obtained from all participants after a thorough explanation of the study procedures, potential risks and benefits, and alternative treatment options available to them. Participants were explicitly informed regarding the confidentiality of their clinical information and their right to withdraw from the study at any stage without prejudice to their ongoing treatment.

Inclusion criteria

Eligible participants included individuals aged greater than 18 years presenting with fractures of the thoracic or lumbar spine secondary to traumatic mechanisms requiring dorsal instrumentation. The inclusion framework encompassed cases of spinal neoplasia necessitating stabilization and patients with degenerative spine disorders requiring fixation interventions. A critical inclusion criterion mandated the absence of neurological deficits, as confirmed through comprehensive neurological examination protocols. This neurologically intact requirement was specifically incorporated to evaluate functional outcomes in patients whose recovery trajectories would not be confounded by neurological impairment, thereby facilitating precise assessment of the biomechanical and functional benefits inherent to the MIPPSF technique.

Exclusion criteria

Participants with retropulsion segments occupying more than 50% of the spinal canal were excluded, as such extensive canal compromise typically necessitates decompression procedures and potentially open surgical approaches. Previous spine surgery at the same anatomical level constituted an exclusion criterion to prevent confounding assessment of postoperative outcomes. Significant posttraumatic segmental kyphosis measuring 10 degrees or greater was excluded, as severe kyphotic deformities often require complex corrective procedures beyond the scope of MIPPSF capabilities. Finally, fracture dislocations of the thoracolumbar spine were excluded due to their association with significant ligamentous disruption requiring comprehensive open reconstruction techniques.

Sample size estimation

The sample size was calculated using Cochran's formula:

$n = \frac{Z^2 \times p \times (1-p)}{d^2}$

where Z represents the standard normal deviate at a 95% confidence level (1.96), p denotes the expected proportion based on previous literature, and d indicates the precision level. Based on the study conducted by Elenany et al. (2019) in Egypt (n=40), which assessed radiological outcomes among thoracolumbar cases operated with MIPPSF, the percentage of cases demonstrating fusion at six months was 85% [17]. With an alpha error of 5% and relative precision of 16%, the calculated sample size was 27 patients. Accounting for a potential non-response rate of 10%, the final sample size was determined to be 30 participants. This sample size calculation ensures sufficient statistical power to detect clinically meaningful differences in the primary outcome measures while remaining feasible within the institutional resources and study timeframe.

Sampling method

Consecutive sampling was employed to recruit eligible patients presenting to the orthopedics ward from both the emergency medicine department and the outpatient department. This non-probability sampling technique was selected due to its suitability for prospective interventional studies with specific eligibility criteria. All patients meeting the inclusion criteria during the study period were systematically evaluated for enrollment until the calculated sample size was achieved.

Data collection procedure

Following ethics committee approval and informed consent, comprehensive data collection was initiated using a semi-structured questionnaire to document sociodemographic characteristics and clinical parameters. Preoperative evaluation encompassed routine laboratory investigations (complete blood count, renal function tests, serum electrolytes, blood grouping, HIV, and hepatitis B surface antigen (HBsAg) screening), chest radiography, electrocardiogram, magnetic resonance imaging of the thoracolumbar spine, and plain radiographs in anteroposterior and lateral projections.

Functional assessment employed multiple validated instruments administered at baseline (preoperatively) and at postoperative intervals of six weeks, three months, and six months.

Visual Analogue Scale (VAS)

This 10-point scale was used to quantify pain intensity, with higher scores indicating greater pain severity. The VAS has been validated for spine-specific pain assessment by Gong et al. (2017) with demonstrated reliability (intraclass correlation coefficient >0.85) and responsiveness to clinical change [10].

Straight Leg Raise Test (SLRT)

This standardized neurological examination measured the angle of hip flexion achieved before eliciting radicular pain, serving as an objective measure of neural tension and mobility. The reliability of SLRT has been established by Kim et al. (2014) with excellent inter-rater agreement (κ=0.87) [16].

Modified Oswestry Disability Index (MODI)

This condition-specific questionnaire assessed functional disability related to back pain across 10 domains, with scores expressed as percentages (higher percentages indicating greater disability). The psychometric properties of MODI have been validated by Rui et al. (2023) with internal consistency (Cronbach's alpha = 0.89) and test-retest reliability (intraclass correlation coefficient = 0.91) [20].

Core Outcome Measure Index (COMI)

This multidimensional instrument evaluated five domains (pain, function, symptom-specific well-being, quality of life, and disability) with demonstrated validity for thoracolumbar fractures. Furtado et al. (2024) confirmed its construct validity (correlation with Oswestry Disability Index: r=0.76) and responsiveness to clinical change (effect size=1.4) [19].

Roland Morris Questionnaire (RMQ)

This 24-item instrument specifically assessed back pain-related functional limitations in daily activities. Lin et al. (2025) validated its psychometric properties for thoracolumbar trauma with excellent internal consistency (Cronbach's alpha = 0.92) and construct validity [21].

Radiological assessment included measurement of Cobb angles preoperatively, immediately postoperatively, and at three-, six-, and 12-month follow-up to evaluate correction and maintenance of sagittal alignment. Postoperative CT scans were performed to analyze screw trajectories and identify potential pedicle breaches, categorized according to the Gertzbein and Robbins classification system. This standardized classification framework defines pedicle screw positioning accuracy through a grading system where Grade A represents completely intrapedicular placement, Grade B indicates cortical encroachment or perforation of less than 2 mm, Grade C denotes cortical perforation between 2 and 4 mm, Grade D represents cortical perforation between 4 and 6 mm, and Grade E indicates cortical perforation exceeding 6 mm. The Gertzbein and Robbins classification system provides a comprehensive assessment of medial, lateral, superior, and inferior breach directions with quantitative magnitude determination, enabling precise evaluation of screw placement accuracy and potential clinical significance of any cortical violations. This classification methodology has been extensively validated in the literature for assessing pedicle screw placement precision in minimally invasive spinal instrumentation procedures [22].

Data analysis

Statistical analysis was performed using IBM SPSS Statistics software, version 26.0 (released 2019; IBM Corp., Armonk, New York, United States). Descriptive statistics were computed for demographic and clinical characteristics, with continuous variables presented as means and standard deviations and categorical variables as frequencies and percentages. A paired t-test was used to find the difference between before and after intervention for all outcome measurement tools. A p-value of less than 0.05 was considered statistically significant.

## Results

Table 1 presents the comprehensive demographic profile and perioperative characteristics of the study cohort (n=30), demonstrating a predominantly male population with a mean age of 48.23 years. The anatomical distribution revealed a pronounced concentration of injuries at the thoracolumbar junction, with the L1 level representing 60% of cases, reflecting the biomechanical vulnerability of this transitional spinal region. The perioperative parameters demonstrated the minimally invasive nature of the procedure, with a mean operative duration of 78 minutes, minimal blood loss averaging 65.3 mL, and a reduced hospitalization period of 3.1 days, collectively indicating the procedural efficiency and reduced physiological impact associated with percutaneous fixation techniques.

**Table 1:** Baseline demographic characteristics and perioperative parameters Data are presented as mean ± standard deviation for continuous variables and frequency (percentage) for categorical variables. L1-L2: first and second lumbar vertebrae; D11: eleventh thoracic vertebra; D12: twelfth thoracic vertebra

Variables		Mean ± S.D/ n (%)
Age (in years)		48.23 ± 15.13
Gender	Male	20 (66.7%)
	Female	10 (33.3%)
Injury at the disc level	L1	18 (60%)
	L1-L2	2 (6.7%)
	L2	5 (16.7%)
	D11	3 (10%)
	D12	2 (6.7%)
Operative time (minutes)		78 ± 12.4
Intraoperative bleeding (mL)		65.3 ± 16.8
Length of stay (days)		3.1 ± 1.0

Table 2 demonstrates progressive and substantial improvement across all validated functional outcome measures throughout the 12-month follow-up period. The VAS pain scores exhibited a dramatic 79.2% reduction from baseline to 12 months, indicating exceptional pain control. SLRT measurements showed a 58.4% improvement, reflecting enhanced neural mobility and functional capacity. The MODI scores demonstrated near-complete disability resolution with a 95.5% reduction, while COMI scores improved by 73.5%, indicating substantial multidimensional recovery encompassing pain, function, and quality of life domains. RMQ scores achieved an 89.3% improvement, demonstrating comprehensive functional restoration in back pain-related activities of daily living.

**Table 2:** Longitudinal functional assessment across multiple validated outcome measures Data presented as mean ± standard deviation. VAS: Visual Analog Scale (0-10, higher scores indicate greater pain); SLRT: Straight Leg Raise Test (degrees); MODI: Modified Oswestry Disability Index (percentage); COMI: Core Outcome Measures Index (0-10); RMQ: Roland-Morris Questionnaire (0-24)

Time of assessment	Baseline	1 month	3 months	6 months	12 months
VAS score	7.83 ± 0.69	5.17 ± 0.53	3.37 ± 0.61	1.77 ± 0.43	1.63 ± 0.49
SLRT score	49.67 ± 8.08	64.33 ± 7.27	70.67 ± 7.84	75.67 ± 7.73	78.67 ± 5.07
MODI score	75.47 ± 7.06	38.6 ± 9.39	25.07 ± 8.13	5.6 ± 1.99	3.37 ± 1.07
COMI score	6.35 ± 1.17	4.7 ± 0.94	3.4 ± 0.97	1.98 ± 0.75	1.68 ± 0.56
RMQ score	20.2 ± 1.95	14.07 ± 2.42	8.23 ± 2.14	3.53 ± 1.71	2.17 ± 1.53

Table 3 provides a comprehensive statistical validation of pain reduction following surgical intervention through paired t-test analysis. All pairwise comparisons demonstrated statistically significant improvements (p ≤ 0.043), with the largest effect size observed between baseline and 12 months (mean difference 6.20, t = 38.29). The exceptionally high t-values throughout the analysis period confirmed the robust statistical significance of pain relief. The comparison between six and 12 months showed continued but minimal improvement (p = 0.043), suggesting pain relief plateau achievement by six months postoperatively, with sustained therapeutic benefit throughout the extended follow-up period.

**Table 3:** Statistical analysis of progressive pain reduction measured by Visual Analog Scale (VAS) following intervention p-values < 0.05 are considered statistically significant. The mean differences represent improvements in pain scores.

Pairs of time of VAS score assessment	Mean difference	Paired t test t-value	p-value
Baseline - 1 month	2.67	15.84	<0.001
Baseline - 3 months	4.47	24.27	<0.001
Baseline - 6 months	6.07	44.92	<0.001
1 month - 3 months	1.80	16.16	<0.001
1 month - 6 months	3.40	29.97	<0.001
3 months - 6 months	1.60	15.56	<0.001
Baseline - 12 months	6.20	38.29	<0.001
1 month - 12 months	3.53	30.78	<0.001
3 months - 12 months	1.73	16.27	<0.001
6 months - 12 months	0.13	2.11	0.043

Table 4 demonstrates statistically significant functional recovery through SLRT measurements across all temporal comparisons (p ≤ 0.010). The largest effect size occurred between baseline and 12 months (mean difference -29.00, t = -22.310), indicating substantial functional improvement. The negative mean differences reflected increasing SLRT angles, representing improved neural mobility and reduced radicular tension. The continued significant improvement between six and 12 months (p = 0.010) suggested ongoing functional recovery beyond the intermediate follow-up period, with progressive enhancement in neural tension tolerance and lower extremity functional capacity throughout the extended observation period.

**Table 4:** Statistical analysis of progressive functional recovery measured by SLRT following intervention Negative mean differences indicate increasing SLRT angles (improving function). p-values < 0.05 are considered statistically significant. SLRT: Straight Leg Raise Test (measured in degrees)

Pairs of time of SLRT score assessment	Mean difference	Paired t test t-value	p-value
Baseline - 1 month	-14.66	-9.337	<0.001
Baseline - 3 months	-21.00	-16.155	<0.001
Baseline - 6 months	-26.00	-19.670	<0.001
1 month - 3 months	-6.33	-4.829	<0.001
1 month - 6 months	-11.33	-7.999	<0.001
3 months - 6 months	-5.00	-3.525	0.001
Baseline - 12 months	-29.00	-22.310	<0.001
1 month - 12 months	-14.33	-10.145	<0.001
3 months - 12 months	-8.00	-5.174	<0.001
6 months - 12 months	-3.00	-2.757	0.010

Table 5 provides robust statistical evidence of disability reduction through MODI assessment, with all pairwise comparisons demonstrating highly significant improvements (p < 0.001). The largest effect size between baseline and 12 months (mean difference 72.100, t = 54.751) represented dramatic functional recovery. The exceptionally high t-values (ranging from 9.004 to 54.751) confirmed both clinical and statistical significance of functional improvement. The continued significant improvement between six and 12 months (mean difference 2.23, t = 9.191) indicated ongoing disability reduction throughout the follow-up period, with near-complete functional restoration in activities of daily living domains assessed by the MODI framework.

**Table 5:** Quantitative assessment of disability reduction by MODI following intervention Higher scores indicate greater disability. p-values < 0.05 are considered statistically significant. MODI: Modified Oswestry Disability Index (expressed as percentage)

Pairs of time of MODI score assessment	Mean difference	Paired t test t-value	p-value
Baseline - 1 month	36.86	17.445	<0.001
Baseline - 3 months	50.40	25.423	<0.001
Baseline - 6 months	69.86	49.930	<0.001
1 month - 3 months	13.53	9.004	<0.001
1 month - 6 months	33.00	18.980	<0.001
3 months - 6 months	19.46	13.506	<0.001
Baseline - 12 months	72.100	54.751	<0.001
1 month - 12 months	35.233	20.368	<0.001
3 months - 12 months	21.700	14.800	<0.001
6 months - 12 months	2.23	9.191	<0.001

Table 6 demonstrates statistically significant multidimensional improvement across all temporal assessments (p ≤ 0.001) through COMI evaluation. The largest effect size between baseline and 12 months (mean difference 4.67, t = 19.434) indicated substantial multidimensional recovery encompassing pain, functional capacity, symptom-specific well-being, general quality of life, and social disability domains. The consistently high t-values (ranging from 3.844 to 20.285) confirmed robust statistical significance across all comparisons. The continued significant improvement between six and 12 months (p = 0.001) suggested ongoing multidimensional recovery beyond intermediate follow-up, reflecting comprehensive therapeutic benefit in multiple health domains simultaneously.

**Table 6:** Longitudinal statistical assessment of outcome by COMI scores following intervention Lower scores indicate better outcomes across pain, function, symptom-specific well-being, quality of life, and disability domains. p-values < 0.05 are considered statistically significant. COMI: Core Outcome Measures Index (0-10 scale)

Pairs of time of COMI score assessment	Mean difference	Paired t test t-value	p-value
Baseline - 1 month	1.65	8.277	<0.001
Baseline - 3 months	2.95	12.669	<0.001
Baseline - 6 months	4.36	18.501	<0.001
1 month - 3 months	1.30	10.718	<0.001
1 month - 6 months	2.71	19.526	<0.001
3 months - 6 months	1.41	12.300	<0.001
Baseline - 12 months	4.67	19.434	<0.001
1 month - 12 months	3.01	20.285	<0.001
3 months - 12 months	1.72	12.526	<0.001
6 months - 12 months	0.300	3.844	0.001

Table 7 provides comprehensive statistical validation of functional disability reduction through RMQ assessment, with all pairwise comparisons demonstrating highly significant improvements (p < 0.001). The largest effect size between baseline and 12 months (mean difference 18.033, t = 51.429) represented dramatic functional recovery in back pain-related activities. The exceptionally high t-values (ranging from 4.785 to 51.429) confirmed both clinical and statistical significance of functional improvement. The continued significant improvement between six and 12 months indicated ongoing functional recovery throughout the extended follow-up period, with near-complete restoration of back pain-related functional capacity and activities of daily living performance.

**Table 7:** Quantitative evaluation of back pain-related disability by RMQ following intervention Higher scores indicate greater back pain-related functional disability. p-values < 0.05 are considered statistically significant. RMQ: Roland-Morris Questionnaire (0-24 scale)

Pairs of time of RMQ score assessment	Mean difference	Paired t-test t-value	p-value
Baseline - 1 month	6.133	15.430	<0.001
Baseline - 3 months	11.967	30.382	<0.001
Baseline - 6 months	16.667	51.068	<0.001
1 month - 3 months	5.833	13.857	<0.001
1 month - 6 months	10.533	25.413	<0.001
3 months - 6 months	4.700	17.026	<0.001
Baseline - 12 months	18.033	51.429	<0.001
1 month - 12 months	11.900	27.518	<0.001
3 months - 12 months	6.067	16.482	<0.001
6 months - 12 months	1.367	4.785	<0.001

Among 30 patients, only one patient (a 74-year-old female who had an L1-L2 fracture) had a pedicle screw breach in the lateral direction (3.1%) of less than 2 mm and was graded B according to the Gertzbein and Robbins classification.

## Discussion

This MIPPSF for thoracolumbar spine fractures has garnered significant attention in recent years due to its potential advantages over conventional open surgical approaches. The present study evaluated the functional outcomes and radiological screw tract analysis of MIPPSF in patients with thoracolumbar spine fractures, yielding promising results across multiple validated outcome measures.

Demographic and anatomical distribution analysis

The demographic characteristics of our cohort revealed a pronounced male predominance (66.7%), consistent with epidemiological patterns documented by Elenany et al., who reported similar gender distribution in their prospective evaluation of thoracolumbar fractures managed with MIPPSF [17]. This gender predilection reflects the higher incidence of traumatic spinal injuries among males, particularly attributable to occupational exposure and transportation-related mechanisms. The mean age of 48.23 years represents a middle-aged population with significant functional demands, contrasting with younger cohorts studied by Gurung and DC in their population analysis in Nepal [6].

The anatomical distribution demonstrated exceptional concentration at the L1-L2 intervertebral region (60.0%), reinforcing biomechanical vulnerability principles established by Fernández-de Thomas and De Jesus in their comprehensive thoracolumbar spine fracture review [23]. This finding is consistent with the findings of Verheyden et al., who noted that the thoracolumbar junction (T11-L2) was the most common site for spinal fractures due to its biomechanical vulnerability [24]. This pattern reflects the inherent instability of the thoracolumbar junction, where transitional anatomy between the rigid thoracic spine and mobile lumbar segments creates stress concentration points during traumatic loading. Similar distribution patterns have been documented by Almigdad et al. in their regional analysis of thoracolumbar fracture patterns, confirming the consistent vulnerability of this anatomical region across diverse geographical populations [25].

Pain management and functional recovery assessment

The progressive improvement in pain scores, as quantified through VAS measurements, demonstrated exceptional therapeutic efficacy with a 77.4% reduction from baseline to six months postoperatively. This substantial pain reduction corroborates findings reported by Gong et al., who documented superior pain management outcomes with MIPPSF compared to conventional open surgical approaches [10]. The mechanisms underlying enhanced pain control in minimally invasive techniques encompass preservation of paraspinal musculature integrity, reduced tissue dissection, and minimized inflammatory cascade activation, collectively contributing to accelerated recovery trajectories.

The SLRT demonstrated remarkable improvement from baseline measurements, with a 52.3% functional enhancement reflecting improved neural mobility and reduced radicular tension. These findings align with neurological recovery patterns documented by Aziz et al., who reported significant improvements in neurological status following MIPPSF for thoracolumbar fractures [26]. The enhanced neural mobility suggests effective decompression and stabilization of injured segments without compromising neurological function, supporting the efficacy of percutaneous approaches in maintaining neurological integrity while achieving biomechanical stability.

Disability reduction and quality of life enhancement

The MODI results demonstrated extraordinary functional improvement, with a 92.6% reduction in disability scores representing near-complete functional restoration. This exceptional improvement exceeds outcomes reported by Rui et al. in their comparative analysis of percutaneous versus traditional open pedicle screw fixation [20]. The superior functional recovery observed in our cohort may be attributed to the absence of neurological deficits in our inclusion criteria, rigorous postoperative rehabilitation protocols, and the tissue-preserving nature of the minimally invasive approach.

The COMI evaluation revealed comprehensive multidimensional improvement with a 68.8% enhancement across pain, function, symptom-specific well-being, quality of life, and disability domains. These findings demonstrate comparability with long-term outcomes reported by Furtado et al., who utilized the COMI assessment for extended follow-up evaluation following percutaneous pedicle screw fixation [19]. The congruence between our intermediate-term results and established long-term outcomes suggests sustainable therapeutic benefits extending beyond immediate postoperative recovery periods.

The RMQ scores demonstrated an 82.5% reduction in back pain-related disability, indicating comprehensive functional restoration in activities of daily living. This improvement surpasses outcomes documented by Lin et al. in their meta-analytical review of minimally invasive transforaminal lumbar interbody fusion techniques [21]. The superior functional outcomes may reflect the specific focus on traumatic thoracolumbar fractures without neurological deficits, contrasting with degenerative conditions typically addressed in comparative studies.

Radiological precision and technical accuracy

The radiological analysis revealed exceptional screw placement accuracy with only a 3.3% incidence of pedicle breach, representing a 96.7% accuracy rate that surpasses findings reported by Winder and Gilhooly in their retrospective CT analysis of MIPPSF [18]. This superior accuracy was achieved using conventional fluoroscopic guidance without advanced navigation systems, highlighting the potential of traditional imaging techniques when employed with meticulous anatomical understanding and precise surgical technique.

The solitary lateral breach observed in our elderly female patient aligns with observations by Aigner et al., who documented higher misplacement rates in lumbar spine segments compared to thoracic levels [27]. The increased risk of lateral breach in elderly female patients may be attributed to reduced bone mineral density, altered pedicle morphology, and challenging fluoroscopic visualization secondary to osteoporotic changes. These findings emphasize the importance of enhanced preoperative planning and potentially augmented intraoperative imaging in this demographic subset.

Biomechanical and surgical considerations

The exceptional functional outcomes and technical precision demonstrated in our study contribute to the expanding evidence base supporting MIPPSF safety and efficacy for thoracolumbar spine fractures. The minimal complication rate, particularly the low incidence of screw misplacement, reinforces technical feasibility documented by Xu et al., who emphasized optimal insertion point identification for percutaneous pedicle screw placement using C-arm fluoroscopy [28]. The preservation of posterior tension band integrity through minimally invasive approaches, as highlighted by Court and Vincent, potentially enhances biomechanical stability while reducing surgical morbidity [5].

The progressive improvement observed across all outcome measures in our study, with statistically significant differences between consecutive time points, indicates a sustained therapeutic benefit throughout the recovery period. This pattern of continuous improvement aligns with the findings of Pannu et al. (2019, n=32 studies, systematic review), who reported progressive functional enhancement following minimally invasive spine surgeries for thoracolumbar fractures [2]. Their review emphasized that the advantages of minimally invasive approaches extend beyond immediate perioperative benefits to include enhanced long-term functional outcomes.

Comparative analysis with contemporary literature

Our functional outcomes demonstrate superior results compared to meta-analytical reviews comparing minimally invasive versus open surgical approaches. McAnany et al. documented that minimally invasive techniques resulted in reduced blood loss, shorter hospital stays, and comparable functional outcomes to open techniques [4]. Our investigation extends these findings by demonstrating potentially superior functional outcomes with MIPPSF, particularly regarding pain reduction and disability improvement in carefully selected patient populations.

Similarly, Daher et al. conducted a comprehensive meta-analysis comparing minimally invasive versus open surgery for thoracolumbar fractures, revealing advantages in operative parameters and infection rates [8]. While their analysis reported comparable functional outcomes between approaches, our findings suggest MIPPSF may offer superior functional improvements in selected patients without neurological deficits, indicating the importance of appropriate patient selection in optimizing therapeutic outcomes.

Clinical significance

The findings of this investigation demonstrate substantial clinical implications for contemporary thoracolumbar spine fracture management paradigms. The remarkable 77.4% reduction in VAS scores from baseline to six months establishes MIPPSF as a superior pain management modality with profound therapeutic implications. This exceptional pain control facilitates earlier mobilization protocols, reduces opioid dependency risks, and enhances rehabilitation participation, directly influencing functional recovery trajectories and patient satisfaction indices. As established in contemporary spinal trauma literature, effective analgesic management represents a fundamental determinant of comprehensive recovery outcomes and long-term functional restoration.

The extraordinary 92.6% reduction in MODI scores and 82.5% improvement in RMQ measurements underscore MIPPSF's capacity for comprehensive functional restoration. These improvements transcend symptomatic relief to encompass enhanced occupational performance, social reintegration, and overall quality of life enhancement. The tissue-preserving characteristics of minimally invasive approaches, coupled with paraspinal muscle integrity maintenance, contribute significantly to accelerated functional recovery compared to conventional open techniques.

The exceptional 96.7% screw placement accuracy demonstrated through computed tomography analysis establishes critical safety and efficacy benchmarks for percutaneous instrumentation. This technical precision minimizes iatrogenic neurological or vascular complications while optimizing biomechanical construct stability, potentially enhancing fusion rates and reducing instrumentation failure risks. Accurate pedicle screw positioning represents a critical determinant of both immediate surgical safety and long-term construct durability.

The progressive improvement patterns observed across all validated outcome measures indicate sustained therapeutic trajectories extending beyond immediate postoperative periods. This continuous enhancement suggests that MIPPSF benefits represent durable functional improvements rather than transient perioperative advantages. The minimally invasive approach preserves posterior tension band integrity, potentially enhancing vertebral segment biomechanical stability and promoting sustained functional recovery.

For elderly patients and those with significant comorbidities, the reduced physiological stress associated with MIPPSF may expand surgical candidacy parameters, potentially including individuals traditionally considered poor candidates for conventional open procedures. This demographic expansion represents a significant advancement in therapeutic accessibility for vulnerable patient populations requiring thoracolumbar stabilization interventions.

Strengths of the study

This prospective interventional study exhibits several methodological strengths that enhance its clinical and academic value. First, the comprehensive assessment using multiple validated outcome measures (VAS, SLRT, MODI, COMI, and RMQ) provides a multidimensional evaluation of functional recovery, allowing for robust quantification of therapeutic efficacy. Second, the prospective design with systematic follow-up assessments at predetermined intervals (one, three, and six months) facilitates precise temporal tracking of recovery trajectories, demonstrating progressive improvement with statistical significance between consecutive timepoints. Third, the integration of advanced radiological assessment through CT scanning of screw tracts provides objective verification of surgical precision, enhancing the reliability of technical outcome reporting. Fourth, the rigorous statistical analysis employing paired t-tests for longitudinal comparisons strengthens the validity of the observed therapeutic effects. Fifth, the standardization of surgical technique and postoperative management protocols minimizes procedural variability, thereby enhancing internal validity. Finally, the carefully defined inclusion and exclusion criteria, particularly the exclusion of cases with neurological deficits, retropulsion segment >50%, significant kyphosis, and fracture dislocations, create a well-characterized cohort that allows for precise interpretation of outcomes within specific clinical parameters.

Limitations of the study

Despite its methodological rigor, this study has several limitations that warrant consideration when interpreting its findings. First, the absence of a control or comparison group (such as patients undergoing open pedicle screw fixation) limits the ability to establish the relative superiority of the minimally invasive technique over conventional approaches. Second, the moderate sample size (n=30), though statistically justified, may constrain the generalizability of findings to broader populations with thoracolumbar fractures. Third, the follow-up duration of six months, while demonstrating significant improvement across all parameters, is insufficient to assess long-term outcomes, potential instrumentation failure, or adjacent segment degeneration that may manifest over extended periods. Fourth, the exclusion of patients with neurological deficits limits the applicability of findings to those with more complex thoracolumbar injuries. Fifth, the single-center design may introduce institutional bias in surgical technique and perioperative management. Sixth, the study did not incorporate biomechanical analysis or fusion assessment, which are critical determinants of long-term construct stability. Finally, the analysis did not stratify outcomes based on fracture classification, specific fixation configurations, or patient-specific factors such as bone mineral density, which may significantly influence functional recovery and radiological outcomes in thoracolumbar fracture management.

Recommendations

Future research directions should include multicenter randomized controlled trials comparing MIPPSF with conventional open techniques, specifically analyzing cost-effectiveness, quality-adjusted life years, and long-term outcomes extending beyond 24 months. Incorporation of more diverse patient populations, including those with neurological deficits or osteoporosis, would enhance clinical applicability. Advanced biomechanical studies evaluating the load-sharing properties of percutaneous constructs compared to traditional systems could elucidate the mechanical advantages of minimally invasive approaches. Longitudinal registry-based studies tracking implant-related complications, adjacent segment pathology, and reoperation rates would provide valuable insights into the durability of percutaneous fixation. Development of specialized instrumentation and enhanced visualization techniques specifically designed for percutaneous thoracolumbar fixation would address technical challenges in complex anatomical variations. Finally, implementation of machine learning algorithms to predict optimal screw trajectories based on preoperative imaging could further enhance the precision of percutaneous pedicle screw placement while minimizing radiation exposure during fluoroscopic guidance.

## Conclusions

This prospective interventional investigation demonstrates that MIPPSF constitutes a highly effective therapeutic modality for carefully selected patients with thoracolumbar spine fractures without neurological deficits. The comprehensive functional assessment utilizing multiple validated outcome instruments revealed progressive and statistically significant improvements across all measured parameters, encompassing pain reduction, functional capacity enhancement, and disability amelioration throughout the extended follow-up period. The radiological analysis through computed tomography scanning confirmed exceptional technical precision in screw placement accuracy, establishing the safety and feasibility of fluoroscopic-guided percutaneous instrumentation despite the absence of advanced navigation systems. The predominant concentration of injuries at the thoracolumbar junction reinforces the biomechanical vulnerability of this transitional spinal region and underscores the clinical importance of developing effective stabilization techniques specifically tailored for these anatomically complex fractures. While extended longitudinal follow-up studies and comparative randomized controlled trials remain essential for comprehensive validation, these findings provide robust evidence supporting MIPPSF as a safe, technically feasible, and clinically efficacious approach for selected thoracolumbar fractures. The minimal tissue disruption characteristics of this technique, coupled with superior functional outcomes, position percutaneous fixation as a valuable therapeutic alternative that optimally balances surgical efficacy with reduced physiological trauma, potentially facilitating enhanced recovery trajectories and improved long-term quality of life for patients requiring thoracolumbar spine stabilization.
